# More for less: Improving the biomass yield of a pear cell suspension culture by design of experiments

**DOI:** 10.1038/srep23371

**Published:** 2016-03-18

**Authors:** Stefan Rasche, Denise Herwartz, Flora Schuster, Natalia Jablonka, Andrea Weber, Rainer Fischer, Stefan Schillberg

**Affiliations:** 1 Fraunhofer Institute for Molecular Biology and Applied Ecology IME, Forckenbeckstraße 6, 52074 Aachen, Germany; 2 Institute for Molecular Biotechnology, Worringerweg 1, RWTH Aachen University, 52074 Aachen, Germany; 3 Dr. Babor GmbH & Co. KG, Neuenhofstraße 180, 52078 Aachen, Germany; 4 Justus-Liebig University Giessen, Institute for Phytopathology and Applied Zoology, Phytopathology Department, Heinrich-Buff-Ring 26-32, 35392 Giessen, Germany

## Abstract

Plant cell suspension cultures are widely used for the production of recombinant proteins and secondary metabolites. One of the most important steps during process development is the optimization of yields by testing different cultivation parameters, including the components of the growth medium. However, we have shown that the biomass yield of a cell suspension culture derived from the pear cultivar *Pyrus communis* cv. Champagner Bratbirne can be significantly improved solely by varying the temperature, inoculum density, illumination, and incubation time. In contrast to medium optimization, these simple physical factors are easily controlled and varied, thereby reducing the effort required. Using an experimental design approach, we improved the biomass yield from 146 g fresh weight (FW)/L to 407 g FW/L in only 5 weeks, simultaneously reducing the costs of goods sold per kg biomass from €125 to €45. Our simple approach therefore offers a rapid, efficient and economical process for the optimization of plant cell suspension cultures.

Plant cell suspension cultures can be used for the production of naturally-occurring secondary metabolites and food additives or the large-scale production of high-value recombinant proteins^1^,^2^,^3^. Cell suspension cultures are advantageous over whole plants because the products are easier to purify and have a more consistent quality, which helps to meet the requirements of good manufacturing practice^4^,^5^. As is the case for other production platforms, cell proliferation and biomass accumulation are important components of process optimization. The growth rate of plant cell suspension cultures depends on multiple factors, including medium composition, pH, light, oxygen supply, shaker speed, temperature, incubation time and inoculum density^6^,^7^,^8^,^9^,^10^,^11^. Typical plant cell culture media, such as Murashige and Skoog (MS) medium or Schenk and Hildebrandt medium, contain up to 20 different inorganic salts as well as organic nutrients and plant hormones^12^,^13^. Some basic salts are found in all media but there are more than 40 different components in total which can be used over a broad concentration range^2^,^5^,^14^. Because different plant cell varieties require unique or at least adapted media for optimal growth, comprehensive screening of all components in all possible combinations is labor intensive and expensive.

The experimental design or design of experiments (DOE) approach is the method of choice for medium optimization because many parameters can be varied simultaneously rather than exhaustively testing one factor at a time, and such approaches can therefore detect interactions between factors that are not clear when single factors are tested individually^6^,^7^,^8^,^9^,^15^,^16^. However, the large number of potential medium components means that even a DOE-supported medium optimization approach would require many experiments. For example, a simple mixture screening design for simplex space allowing the initial testing of 20 different factors at high and low levels would still require at least 65 individual experiments.

The growth of cell cultures can be improved not only by medium optimization but also by the analysis and modification of simple physical cultivation factors such as light intensity, shaker speed or temperature. The number of relevant factors that must be tested in combination is much lower than typically required for medium optimization, thus reducing the complexity of the experimental design and the effort required for screening.

We analyzed the impact of four of the most easily controlled physical factors (light, temperature, incubation time and inoculum density) on the biomass yield of a pear suspension cell culture by modelling the design space using a DOE approach. This model enabled us to improve the biomass yield 3-fold, and reduced the cost of goods sold (COGS) by the same order, without any attempt to optimize the culture medium. This approach therefore provides a simple and efficient strategy to improve the performance of plant cell suspension cultures and it is likely that the same method could be applied in other cell-based production platforms to increase productivity and reduce the production costs.

## Results

The pear cell culture was established from *Pyrus communis* cv. Champagner Bratbirne fruit tissue. There was no information available about the optimization of growth conditions for these cells, so we decided to grow them using the conditions described for the well-characterized tobacco cell line BY-2 (Bright Yellow 2). Using these unmodified conditions, fermentation produced a fresh cell weight of 146 g/L in 7 d, whereas BY-2 cell suspension cultures can produce 350 g fresh weight (FW)/L biomass over the same period and under the same conditions^17^. We therefore investigated whether any of the easily-controllable and variable factors light, temperature, incubation time and inoculum density had a significant impact on the accumulation of biomass and whether varying these factors could achieve biomass accumulation similar to BY-2 cells.

To assess these four factors, we set up a IV-optimal response surface method (RSM) comprising 35 runs using Design Expert v.8.04 (Supplement 1). We anticipated that at least the temperature and incubation time would have a quadratic effect on biomass accumulation and therefore used a quadratic design model. The factor ranges, types and levels are shown in Table 1. We cultivated 50-ml aliquots of cells in 100-ml Erlenmeyer flasks under the conditions recommended in the RSM plan (Supplement 1). All aliquots were inoculated from the same source culture to ensure the basic conditions were always equivalent. The fresh cell weight was measured as the output or response.

**Figure 1:**
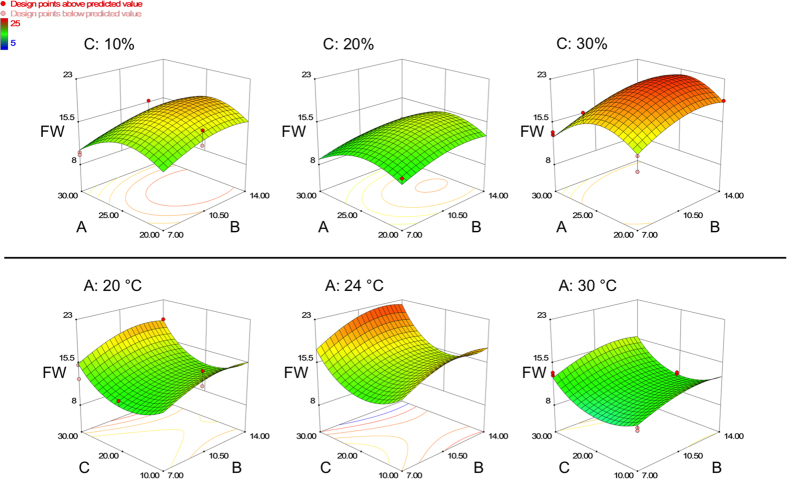
Response surface model for biomass accumulation. Three-dimensional response surface model graphs showing the impact of temperature (**A**, °C), inoculum density (**C**, % (v/v)) and incubation time (**B**, days) on the fresh cell weight yield (FW, grams) of the pear cell suspension culture (50 ml culture volume). Upper row: Fresh cell weight yield shown as a function of incubation temperature (**B**) and incubation time (**A**), while the inoculum density is set constant at three different levels: 10%, 20% and 30%. Lower row: Fresh cell weight yield shown as a function of inoculum density (**C**) and incubation time (**A**), while the incubation time is set constant at three different levels: 20 °C, 24 °C and 30 °C. All cells were grown under illumination (16-h photoperiod).

**Figure 2:**
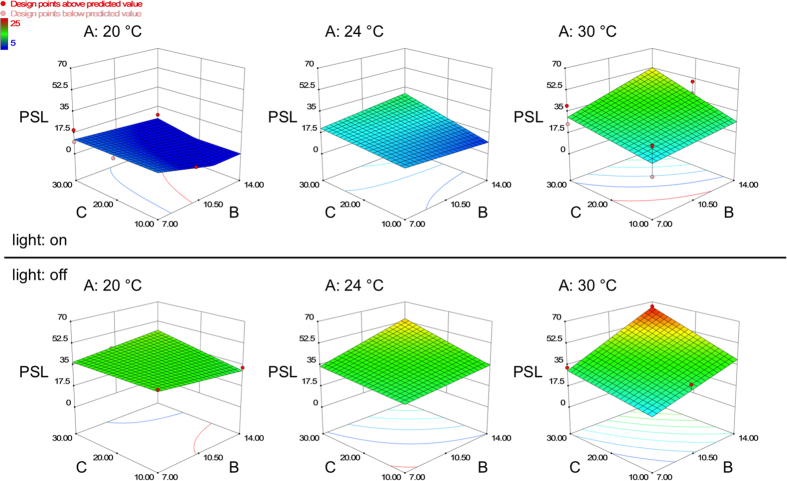
Response surface model for PSL content during cultivation. Three-dimensional response surface model graphs showing the impact of temperature (**A**, °C), inoculum density (**C**, % (v/v)) and incubation time (B, days) on the percentage content of potential polysaccharides and/or lipids (PSL) secreted by the pear cells (50 ml culture volume). The PSL content in relation to the pear cell biomass after centrifugation is shown as a function of inoculum density (**C**) and incubation temperature (**B**), while the incubation time is set constant at three different levels: 20 °C, 24 °C and 30 °C. Upper row: under illumination (16-h photoperiod), lower row: no illumination.

Analysis of variance (ANOVA, Table 2) showed that all four of the main factors had a significant impact on biomass yield. Light was essential for the growth of the pear cells and we therefore observed only a slight increase in biomass yield in the experiments without illumination (data not shown). Furthermore, three interactions between the main factors were found to be significant, and the interaction between light and inoculum density was found to be highly significant. The predicted R^2^ value was in reasonable agreement with the adjusted R^2^ value and the lack of fit test was not significant, confirming the significance of the model (Table 3). Among the four factors we tested, the inoculum density and incubation temperature had the strongest influence on biomass accumulation (Fig. 1). The conditions that supported the highest biomass yield were 30% (v/v) inoculum density, 24 °C incubation temperature and 14 days cultivation time, with a predicted yield of 445 g FW/L. To ensure that the increase in biomass yield is not affected by osmolality driven erratic water uptake, we performed additional experiments measuring the osmolality at day-0 (167 mOsmol/kg) and at day-10 of the cultivation period, following the cultivation conditions given by the design (Table 1). On average, we obtained a level of 23 ± 4 mOsmol/kg at day-10, independent on the culture conditions (data not shown). The low osmolality at the end of the cultivation period will lead to an increased water uptake by the cells, but as the values are quite similar, the impact on the outcome of the DoE is negligible. During the experiment (and especially after centrifuging the cells) we observed the formation of a white, viscous substance with a lower density than the cells. We assumed that the substance was composed of polysaccharides and/or lipids secreted by the cells during cultivation. The quantity of this “potential polysaccharides or lipids” (PSL) component relative to the biomass was strongly dependent on the cultivation conditions (Fig. 2). Therefore we decided to measure the PSL content by volume and to set this as a further response in addition to the cell fresh weight, using the same RSM design for the evaluation. The ANOVA (Table 4) showed that all the main factors except incubation time had a significant impact on the formation of PSL. There were also significant interactions among all the main factors, and the interaction between light and temperature was highly significant. The predicted R^2^ value was in reasonable agreement with the adjusted R^2^ value (Table 5) even though both values were lower than those obtained for the biomass prediction model. These differences were most likely caused by the measurement method. The PSL volume was determined by reading gradations on the centrifugation tube, whereas the biomass yield was determined more precisely by weighing. Even so, the model quality and the non-significant lack of fit test ensured the significance of the model. The conditions supporting the highest biomass yield (inoculum density 30% (v/v), incubation temperature 24 °C, 16-h photoperiod and 14-d cultivation time), were predicted to yield a PSL content of 30%.

To reduce the PSL content, we used the numerical optimization function of Design Expert to find conditions under which the biomass yield and PSL content were in reasonable agreement. The optimization function allows certain goals and restrictions to be set for each significant factor and each response factor. We therefore set the goals to minimize the PSL content and simultaneously to maximize the biomass yield. No goals or restrictions were set for the other factors. We picked the two most suitable solutions from the 60 suggested by the software (Table 6) and tested the model described above. Six replicate cultures representing each solution were inoculated from the same source culture to ensure the basic conditions were always equivalent. In both solutions (S), the yields we achieved exceeded the predicted yields (S3: 366 g FW/L predicted, 407 g FW/L achieved; S4: 336 g FW/L predicted, 392 g FW/L achieved) whereas the PSL content was as predicted (S3: 5% PSL) or lower (S4: 12.5% PSL predicted, 5% PSL achieved). We used solution S3 for further production because higher yields were achieved with a lower PSL content, and it included a more suitable production and cultivation interval. The increased biomass yield of 407 g FW/L compared to the initial yield of only 146 g FW/L resulted in a 3-fold reduction of the COGS per kg biomass (Supplement 2).

## Discussion

Modern statistical approaches that were initially developed for agricultural experiments^12^,^18^ are nowadays used in many different research areas^19^. The power of DOE methods is not only their ability to predict responses such as biomass yield or the accumulation of secondary metabolites^20^,^21^, but also to provide a more holistic view of the cultivation process thus offering insight into the underlying mechanisms^11^,^22^,^23^.

We found that biomass accumulation in a pear cell suspension culture was significantly influenced by light, temperature, incubation time, and inoculum density. This was not unexpected, but using experimental designs we were able to generate a predictive model that was able to improve the yield of the culture from 146 to 407 g FW/L, simultaneously reducing the COGS from €125 to €45 per kg biomass (Supplement 2). Furthermore, the same model was used to reduce the percentage of unwanted byproducts (PSL) secreted by the pear cells during cultivation to 5% (v/v). This was achieved by varying only the physical culture conditions, combining this approach with medium optimization could potentially increase the yield even further. Depending on the cell type and cultivation conditions, biomass yields of up to 500 g FW/L have been reported^24^. However, we have already reached more than 80% of this target just by optimizing the physical cultivation conditions. It is unlikely that the intense effort required to optimize the cultivation medium would be worth pursuing if the further yield increase would not exceed 20%.

Unlike previous studies focusing on particular secondary metabolites or heterologous protein expression, the objective of our investigation was to increase the biomass yield of a novel pear cell suspension culture, because ethanol extracts from these cells are used as an ingredient for products in the cosmetics industry (https://de.babor.com/news/456/babor-spa). However, the amount of PSL that forms during fermentation also depends on the cultivation conditions, and this interesting property will be investigated in more detail in future experiments. In conclusion, we have developed a simple but effective way to improve the biomass yield of an uncharacterized plant suspension cell culture. With little effort, we were able to increase the biomass yield significantly while simultaneously reducing the COGS, proving the power of experimental design to improve the yields of industrially-relevant plant cell cultures.

## Materials and Methods

### Generation of the pear cell suspension culture and initial cultivation conditions

Fruit tissue from the pear cultivar *Pyrus communis* cv. Champagner Bratbirne (kindly provided by Dr. Babor GmbH, Aachen, Germany) was surface sterilized by dipping in 70% (v/v) ethanol and incubating for 10 min in 5% (v/v) sodium hypochloride. The tissue was then rinsed three times with sterile water before transferring to 20 ml AA medium^25^ in a 50-ml Erlenmeyer flask. After incubation for 2 months (26 °C, 140 rpm, 16-h photoperiod, 96 μE/m^2^/s) the resulting callus tissue was incubated for a further 3 months on plates of solid AA medium (26 °C, 16-h photoperiod, 96 μE/m^2^/s) with monthly subculturing on solid fresh AA medium. Cell suspensions were established by resuspending 2-cm callus pieces in liquid 8p2c medium^26^ , and subculturing weekly by transferring 30–90% (v/v) of the culture into 20 ml fresh liquid medium and incubating at 26 °C, 140 rpm with a 16-h photoperiod (96 μE/m^2^/s). Once the cell suspension culture was established, the medium was changed to MS medium^26^ and the cells were further subcultured at 7-d intervals by transferring 20% (v/v) into fresh liquid medium. All experiments related to the optimization of the culturing conditions were done one year after the pear cell suspension culture has been established to avoid inconsistencies in regards to cell growth or growth behaviour.

### Determination of fresh cell weight

The fresh cell weight was determined by transferring the entire culture volume (50 ml) into 50-ml falcon tubes and centrifuging at 3,000 × g for 10 min at 25 °C using a centrifuge equipped with a swing-out rotor. The supernatant was discarded and the mass of the remaining cell pellet was determined. All values for biomass yields presented in the result section are final biomass concentrations (gram fresh weight per liter), including the biomass of the inoculum.

### Design of experiments

The biomass yield of the pear cell suspension culture under different cultivation conditions was predicted using a response surface model with IV-optimal design, generated using Design Expert v.8.0.4 (Stat-Ease Inc., Minneapolis, USA). We analyzed temperature, inoculum density and incubation time as discrete numeric factors, whereas light was analyzed as a nominal categoric factor (on/off). The factor ranges are listed in Table 1. A quadratic model design with IV-optimality comprising 35 runs (14 model points, 16 replicates and 5 runs to determine lack of fit) was used to generate the model.

## Additional Information

**How to cite this article**: Rasche, S. *et al*. More for less: Improving the biomass yield of a pear cell suspension culture by design of experiments. *Sci. Rep.* **6**, 23371; doi: 10.1038/srep23371 (2016).

## Supplementary Material

Supplementary Information

## Figures and Tables

**Table 1:** Overview of factors and factor levels used in the response surface model.

**Factor**	**Name**	**Unit**	**Type**	**Level 1**	**Level 2**	**Level 3**
A	Temperature	°C	Discrete	20	26	30
B	Incubation time	d	Discrete	7	10	14
C	Inoculum density	% (v/v)	Discrete	10	20	30
D	Light (16-h photoperiod)	μE/m^2^/s	Nominal	0	96	–

**Table 2:** Factors and factor interactions used to predict biomass accumulation.

**Factor**	**F-value**	**p-value**
Model	89.30	<0.0001
A (temperature)	1.91	0.1810
B (incubation time)	59.86	<0.0001
C (inoculum density)	126.92	<0.0001
D (light)	561.77	<0.0001
AD	10.03	0.0045
BD	5.69	0.0262
CD	37.95	<0.0001
A^2^	26.65	<0.0001
B^2^	6.40	0.0191
C^2^	40.02	<0.0001

A reduced quadratic model was used to analyze the data. Factors showing a significant influence on the biomass yield were preselected by automated backward selection with a p-value threshold of 0.100. Factors with a p-value > 0.05 were removed manually, except those needed to maintain the model hierarchy. A p-value of 0.05 indicates a significance (alpha) level of 5%.

**Table 3:** Model characteristics to ensure significance (biomass model).

**Parameter**	**Value**
R^2^	0.9760
Adjusted R^2^	0.9650
Predicted R^2^	0.9460
Lack of fit	0.1184

**Table 4:** Factors and factor interactions used to predict the accumulation of PSL.

**Factor**	**F-value**	**p-value**
Model	24.06	<0.0001
A (temperature)	30.61	<0.0001
B (incubation time)	7.25	0.0130
C (inoculum density)	23.93	<0.0001
D (light)	73.87	<0.0001
AB	23.56	<0.0001
AC	4.39	0.0473
AD	25.68	<0.0001
BC	6.74	0.0161
BD	5.59	0.0268

A reduced two-factor interaction model was used to analyze the data. Factors showing a significant influence on the amount of PSL were preselected by automated backward selection with a p-value threshold of 0.100. Factors with a p-value > 0.05 were removed manually, except those needed to maintain the model hierarchy. A p-value of 0.05 indicates a significance (alpha) level of 5%.

**Table 5:** Model characteristics to ensure significance (PSL model).

**Parameter**	**Value**
R^2^	0.9040
Adjusted R^2^	0.8664
Predicted R^2^	0.7891
Lack of fit	0.3372

**Table 6:** Optimized conditions suggested by Design Expert.

**Solution**	**A**	**B**	**C**	**D**	**Yield (g FW/L)**	**PSL content (%)**	**COGS (€/kg)**
S0	26	7	20	on	146	n.d.	125
S3	23	13	10	on	407 ± 10/366*	5 ± 1/5*	45
S4	23.5	9	10	on	392 ± 30/336*	5 ± 0/12.5*	47

Several different solutions were suggested by Design Expert, and the two best examples (S3, S4) are shown here compared to the starting conditions (S0). Values for yield and PSL content marked in italic and with an asterisk represent the predicted values from Design Expert. The first value represents the measured yield/content. See Supplement 2 for details regarding the COGS calculation. n = 6 biological replicates

A: temperature; B: incubation time; C: inoculum density; D: light.
