# Supplementary material for: More for less: Improving the biomass yield of a pear cell suspension culture by design of experiments
Source: Sci Rep. 2016 Mar 18;6:23371. doi: 10.1038/srep23371 (PMC4796904; doi:10.1038/srep23371)
Supplement: Supplementary Information [file srep23371-s1.doc]

**Supplementary Information**

**More for less: Improving the biomass yield of pear cell suspension culture by design of experiments**

Stefan Rasche^1*^, Denise Herwartz^1^, Flora Schuster^2^, Natalia Piotrzkowski^1^, Andrea Weber^3^, Rainer Fischer^1,2^, Stefan Schillberg^1,4^

^1^ Fraunhofer Institute for Molecular Biology and Applied Ecology IME, Forckenbeckstraße 6, 52074 Aachen, Germany

^2^ Institute for Molecular Biotechnology, Worringerweg 1, RWTH Aachen University, 52074 Aachen, Germany

^3^ Dr. Babor GmbH & Co. KG, Neuenhofstraße 180, 52078 Aachen, Germany

^4^ Justus-Liebig University Giessen, Institute for Phytopathology and Applied Zoology, Phytopathology Department, Heinrich-Buff-Ring 26-32, 35392 Giessen, Germany

*Correspondence (Tel +49241 6085 12321; fax +49 241 6085 50007; e-mail: stefan.rasche@ime.fraunhofer.de; address: Fraunhofer Institute for Molecular Biology and Applied Ecology IME, Forckenbeckstraße 6, 52074 Aachen, Germany)

**Supplementary information 1: Summary of the RSM plan.**

| Standard order | Run order | Factor A: temperature (°C) | Factor B: incubation time (d) | Factor C: Inoculum density (% v/v) | Factor D: light (on/off) |
| --- | --- | --- | --- | --- | --- |
| 26 | 1 | 30 | 7 | 30 | 1 |
| 17 | 2 | 20 | 10 | 10 | 1 |
| 28 | 3 | 20 | 14 | 30 | 1 |
| 29 | 4 | 20 | 14 | 30 | 1 |
| 16 | 5 | 20 | 10 | 10 | 1 |
| 4 | 6 | 20 | 14 | 10 | 0 |
| 18 | 7 | 26 | 10 | 10 | 1 |
| 23 | 8 | 30 | 14 | 20 | 1 |
| 21 | 9 | 26 | 7 | 20 | 1 |
| 14 | 10 | 30 | 14 | 30 | 0 |
| 10 | 11 | 30 | 7 | 30 | 0 |
| 24 | 12 | 20 | 7 | 30 | 1 |
| 8 | 13 | 26 | 10 | 20 | 0 |
| 15 | 14 | 30 | 7 | 10 | 1 |
| 20 | 15 | 20 | 7 | 20 | 1 |
| 2 | 16 | 30 | 10 | 10 | 0 |
| 25 | 17 | 26 | 7 | 30 | 1 |
| 12 | 18 | 20 | 10 | 30 | 0 |
| 1 | 19 | 20 | 7 | 10 | 0 |
| 9 | 20 | 26 | 10 | 20 | 0 |
| 11 | 21 | 20 | 10 | 30 | 0 |
| 6 | 22 | 26 | 10 | 20 | 0 |
| 7 | 23 | 26 | 10 | 20 | 0 |
| 22 | 24 | 30 | 14 | 20 | 1 |
| 13 | 25 | 30 | 14 | 30 | 0 |
| 19 | 26 | 26 | 14 | 10 | 1 |
| 3 | 27 | 30 | 10 | 10 | 0 |
| 27 | 28 | 30 | 10 | 30 | 1 |
| 5 | 29 | 20 | 14 | 10 | 0 |
| 30 | 30 | 30 | 7 | 30 | 1 |
| 31 | 31 | 20 | 7 | 10 | 0 |
| 32 | 32 | 30 | 7 | 30 | 0 |
| 33 | 33 | 26 | 14 | 10 | 1 |
| 34 | 34 | 20 | 7 | 30 | 1 |
| 35 | 35 | 30 | 7 | 10 | 1 |

**Supplementary information 2: Calculation of the cost of goods sold.**

To estimate the COGS per kg fresh biomass, all relevant factors were taken into account including personnel and material costs. The costs for running smaller machines (e.g. pumps, electronic pipettes) and non-process related water are covered by the personnel overheads. The personnel costs were calculated based on the average of a post-doctoral researcher and a technician, resulting in an hourly cost of 72.4 € including overheads. * Average energy consumption for one shaker incubator: 0.5 kW

|  | Costs (€)/unit |  | | 12.5-L scale | | 25-L scale | | 50-L scale | | 100-L scale | |
| --- | --- | --- | --- | --- | --- | --- | --- | --- | --- | --- | --- |
| **Medium preparation** |  |  | | Units | Costs (€) | Units | Costs (€) | Units | Costs (€) | Units | Costs (€) |
| Chemicals and water | 3 |  | | 12.5 | 37.5 | 25 | 75 | 50 | 150 | 100 | 300 |
| Working hours | 72.4 |  | | 0.25 | 18.1 | 0.25 | 18.1 | 0.5 | 36.2 | 0.5 | 36.2 |
| Sterile filter | 20 |  | | 1 | 20 | 1 | 20 | 2 | 40 | 4 | 80 |
|  |  |  | |  |  |  |  |  |  |  |  |
| **Production** |  |  | |  |  |  |  |  |  |  |  |
| Single-use disposable reactor | 45 |  | | 5 | 225 | 10 | 450 | 15 | 675 | 20 | 900 |
| Working hours | 72.4 |  | | 0.81 | 58.9 | 1.63 | 117.9 | 2.4 | 176.9 | 3.3 | 235.6 |
| Energy consumption* | 0.08/h |  | | 168 | 13.4 | 336 | 26.9 | 504 | 40.3 | 672 | 53.8 |
|  |  |  | |  |  |  |  |  |  |  |  |
| **Harvest** |  |  | |  |  |  |  |  |  |  |  |
| Filter | 2 |  | | 5 | 10 | 10 | 20 | 15 | 30 | 20 | 40 |
| Working hours | 72.4 |  | | 1 | 72.4 | 1.5 | 108.6 | 2 | 144.8 | 2.5 | 181 |
|  |  |  | |  |  |  |  |  |  |  |  |
| **Solutions** |  |  | |  |  |  |  |  |  |  |  |
|  |  |  | |  |  |  |  |  |  |  |  |
| S0 | Biomass yield (kg/L) | |  | | 0.146 |  | 0.146 |  | 0.146 |  | 0.146 |
|  | Cost (€/kg biomass) | | | | 250 |  | 229 |  | 177 |  | 125 |
|  |  | |  | |  |  |  |  |  |  |  |
| S3 | Biomass yield (kg/L) | |  | | 0.407 |  | 0.407 |  | 0.407 |  | 0.407 |
|  | Cost (€/kg biomass) | | | | 90 |  | 82 |  | 64 |  | 45 |
|  |  | |  | |  |  |  |  |  |  |  |
| S4 | Biomass yield (kg/L) | |  | | 0.392 |  | 0.392 |  | 0.392 |  | 0.392 |
|  | Cost (€/kg biomass) | | | | 93 |  | 85 |  | 66 |  | 47 |
|  |  | |  | |  |  |  |  |  |  |  |
